# Effectiveness of the combined LSPPDM and simulation teaching model in neonatal nursing intern training

**DOI:** 10.3389/fmed.2025.1635264

**Published:** 2025-11-10

**Authors:** Xiaoshan Huang, Xiaoyan Ye, Huilan Yang, Yuexiang Zhang

**Affiliations:** Department of Neonatal Intensive Care Unit, The First Affiliated Hospital of Xiamen University, School of Medicine, Xiamen University, Xiamen, China

**Keywords:** Learn-See-Practice-Prove-Do-Maintain, simulation teaching methods, self-directed learning ability, humanistic caring ability, neonatal nursing

## Abstract

**Objective:**

To explore the effects of applying the Learn–See–Practice–Prove–Do–Maintain (LSPPDM) learning model in combination with simulation-based teaching methods to neonatal nurse teaching practices.

**Methods:**

This is a historical controlled non-randomized quasi-experimental study. The combination LSPPDM–simulation teaching model was officially implemented into the study hospital’s nursing internship educational program in 2023. A control group of 72 interns received instruction by conventional methods between August and December 2022; an observation group of 71 interns received instruction via the LSPPDM–simulation teaching combination model between May 2023 and April 2024. The self-learning ability scale, humanistic care ability evaluation scale, Core Competency Scale for Registered Nurses in China, and final exam scores of the two groups were compared.

**Results:**

The observation group scored significantly higher than the control group with respect to the following aspects: autonomous learning (70.18 ± 7.11 vs. 66.45 ± 8.64, *p* = 0.001), care ability (127.12 ± 4.23 vs. 121.28 ± 11.16, *p* = 0.001), nursing core competence (163.33 ± 21.55 vs. 144.63 ± 19.09, *p* = 0.001), final examination (82.36 ± 3.35 vs. 79.09 ± 6.87, *p* = 0.001), and satisfaction with teaching methods (12.03 ± 0.56 vs. 9.34 ± 0.35, *p* < 0.001).

**Conclusion:**

This study demonstrates that the integration of LSPPDM with simulation-based teaching significantly enhances the self-directed learning, humanistic care, and core competencies of nursing students during their neonatal clinical rotations, offering a novel model for pediatric nursing education. Future research should include multicenter, large-sample, randomized controlled trials to further validate the effectiveness and scalability of this approach.

## 1 Introduction

To address evolving healthcare needs, pediatric nursing education should cultivate both technical competencies and the ability to independently provide compassionate care. The current teacher-centered pediatric nursing instructional model results in insufficient self-directed learning abilities and a weak sense of humanistic care among students and fails to meet the objectives of modern nursing education (1). Therefore, exploring novel teaching models that enhance students’ comprehensive competencies is a vital direction for nursing education reform.

The Learn–See–Practice–Prove–Do–Maintain (LSPPDM) training model, which emphasizes self-directed learning, situational simulation, and a sense of achievement, is an efficient method for creating skills-based learners (2, 3). This approach enhances learning efficiency and cultivates students’ self-directed learning, clinical operation, and problem-solving skills by defining clear learning objectives and strengthening the integration of theory and practice. Compared with traditional teacher-centered models, LSPPDM places greater emphasis on active student participation and practical reflection, providing robust support for the development of skilled professional (4, 5).

Moreover, simulation-based teaching methods utilize high-fidelity simulators and clinical scenarios to provide students with a safe, risk-free learning environment (6, 7). This method bridges the often-present gap in traditional teaching between theory and practice, allowing students to reduce psychological pressure, boost their confidence, and improve their coping skills through repeated practice (8). Simulation-based teaching has achieved outstanding results in numerous clinical teaching settings and has become an indispensable teaching tool in nursing education (9).

Currently, insufficient self-directed learning ability and a weak sense of humanistic care are particularly prominent issues among intern nurses in neonatal departments (10–13). Combining the LSPPDM model with simulation-based teaching can effectively stimulate learning motivation and enhance self-directed learning abilities and humanistic care awareness among nursing students by defining clear learning objectives and presenting stronger situational simulation and opportunities for practice, thereby significantly improving comprehensive competencies and teaching outcomes among intern nurses (14). However, few research reports currently exist on the effectiveness of combining these two methods, and their combined practical value requires further validation.

Accordingly, the teaching content of this study refers to instruction that addresses pediatric nursing learning requirements, this study investigates the effectiveness of combining the LSPPDM model with simulation-based teaching methods in the training of intern nurses in neonatology. The study evaluates the comparative impact on intern nurses’ self-directed learning ability, humanistic care competency, core neonatal nursing skills, and academic performance of a traditional teaching model versus the combined LSPPDM–simulation teaching approach, with the goal of providing a scientific basis and practical reference for nursing education reform.

## 2 Research participants and methods

### 2.1 Sample

This is a historical controlled non-randomized quasi-experimental study. The effect size of the preliminary study was 0.5, with *α* = 0.05 and a test power of 0.80, indicating a requirement of at least 64 cases per group. The instructional method combining LSPPDM with simulation-based teaching to train interns was officially implemented in the study hospital in 2023. Due to varying internship durations, convenience sampling was used to group participants based on their internship periods. A random number table method was used to randomly assign neonatal nursing interns to the observation group and control group, ensuring balanced and comparable baseline data between the two groups. All participants had completed basic medical coursework, occupied similar academic levels, and scored above 80 on operational skills assessments.

Due to teaching schedules and ethical constraints, concurrent random grouping could not be achieved. Seventy-two neonate student nurses working as interns between August and December 2022 were included in the control group; 71 student nurses working as interns at the department between May 2023 and April 2024 were included in the observation group. Conventional teaching methods were applied in the control group, and the combined LSPPDM–simulation teaching model was adopted in the observation group. Teaching was conducted on the condition that the teaching materials involved, total lecturing hours, teaching schedule, and teacher resources were the same for both groups. Inclusion criteria were as follows: (1) a planned total internship duration ≥8 months; and (2) completion and passing of fundamental medical curriculum studies and exams. Exclusion criteria were as follows: (1) off-duty for >1 week for any reason; (2) unwillingness to participate in the study; and (3) internship terminated ahead of schedule.

Objective Structured Clinical Examinations (OSCEs) were scored independently by two examiners unaware of the groupings, thus achieving single blindness. Students were informed of the purpose of the study when completing the questionnaire.

### 2.2 Methods

Conventional teaching methods were adopted in the control group. During class, teachers applied conventional teaching methods to explain and demonstrate operational skills individually, based on prescribed teaching content. Students completed self-directed exercises as instructed by teachers. Teachers conducted group practice on neonatal morning care to improve student nurses’ operational skills and enhance their sense of self-efficacy. Unified theoretical examinations at varying stages were taken after each class.

A training team that comprised four neonatal pediatricians and four supervising nurses, all of whom were hospital-level training teachers, was organized by the nursing department. In addition, a senior nursing expert was included to provide team guidance. The team collectively discussed the prospective instructional content using reference materials and selected and compiled case teaching samples primarily for common topics, such as first aid for premature infants, neonatal asphyxia, and neonatal pneumonia. Teaching was divided into six aspects: learning, observation, practice, verification, operation, and maintenance.

The remaining months were mainly devoted to practice. First, the actual clinical operation process was demonstrated for the interns, and key and difficult points in the process were explained. After the interns had obtained the target skills, they worked independently, conducting a practical exercise every 5 weeks to address knowledge points, reflect on forgotten points, and strengthen their memories. The research team revised, organized, and finally determined the combination teaching method based on expert suggestions (Table 1).

**Table 1:** Teaching design finalization of LSPPDM fusion scenario simulation teaching method in observation group.

Teaching objectives	Knowledge goal	Memorize the common health problems of children ‘s physical, psychological and social needs and the nursing points of common diseases and the basic knowledge of health education.		
	Ability goal	Learn to use the theoretical knowledge of pediatric nursing, and correctly use the relevant scales commonly used in pediatric nursing; accurately determine the common diseases of children and infants, and implement preliminary nursing.		
	Quality goal	Cultivate students’ good teamwork spirit; cultivate students’ good thinking mode and exploration mode; cultivate students’ good professional ethics quality and independent learning ability.		
Teaching Content	It mainly includes teaching materials, other auxiliary teaching materials, props for scenario simulation, laboratory exercises and other teaching contents, involving the learning of the combination of theory and practice of common diseases and frequently-occurring diseases in children and infants.			
Teaching session	Sessions	Teaching methods	Teacher Activity	Student Activity
	Learning	Lecture + Scenario Simulation (twice a week)	Distribution of relevant sitcom scripts prepared before class; platform release: teaching PPT, test question bank.	Two days before class, role-playing, experience situational simulation feelings, arouse interest in learning.
	Observe	Video lectures (twice a week)	Produces relevant teaching videos.	Identify the learning objectives and grasp the knowledge points.
	Exercise	Performing simulator exercises + situational simulation teaching	Focus on explaining the doubts and difficulties; distribute the scripts of relevant scenarios, and complete the relevant clinical operations.	Following the teacher to explain, grasp the key points; with the help of props, role-playing, careful experience in the scene, group cooperation, design solutions.
	Certified	Summary	Summarize Difficulties; Feedback Interns’ Classroom Performance.	Sort out knowledge points and form a mind map; evaluate the recent learning effect.
	Practice	Actual Clinical Operations	Demonstrate the actual clinical operation process for interns, explain the key difficulties in the process.	Following the teacher ‘s explanation, grasp the key points.
	Maintenance	After-school timed exercise	Organize interns regularly to practice; evaluate interns’ performance.	Sort out knowledge points, reflect on forgetting, and strengthen memory.

A combination of LSPPDM and simulation teaching methods was adopted in the observation group. First, a review of relevant literature on LSPPDM and simulation teaching methods, both domestically and internationally, was conducted. Based on actual teaching needs, a preliminary plan was formulated for the combination of LSPPDM and simulation teaching methods. At the end of instruction, students’ comprehensive abilities—including care ability; child healthcare; basic and critically ill pediatric nursing; childhood nutrition and related disease nursing; infectious and neonatal disease nursing; and nursing of diseases of the eight major systems—were tested to confirm they had met requirements.

In the first and second months of the course, instruction focused on learning, observation, and practice. Students were informed of the teaching content in the first week of the course and divided into seven groups, and different scenes were established. Scene simulation was distributed by drawing lots, and students voluntarily chose acting roles within their group. On Monday and Wednesday of the first week of instruction, the teacher took the mornings to explain any relevant knowledge. After the explanation, the routine nursing process of neonate delivery in the study hospital was simulated to stimulate student interest and so that students could understand the disposal methods of different events. Students were then organized to view relevant theoretical teaching videos in the afternoon to consolidate the theoretical content. In Weeks II and IV, scripts of relevant scenarios were issued, and students were organized to perform deeper scenario simulation exercises based on simulators to consolidate and apply the knowledge they had obtained. Weekly tests to examine students’ knowledge were fixed on Fridays. During the study period, after the completion of each day’s instruction, students were asked to summarize key and difficult points, sort out knowledge points, and form a mind map.

### 2.3 Information collection

The age, gender, place of residence, sibling status, educational level, socioeconomic status, reasons for choosing nursing, internship duration, and degree of dedication to the nursing specialty were collected. The learning situation of the two groups was evaluated using self-rated dedication to the profession. Learning outcomes were assessed with the validated Self-Learning Ability Scale for Nursing Students, the Chinese Caring Ability Inventory, the Chinese Registered Nurse Core Competence Scale, and a final examination. A teaching satisfaction evaluation form was issued to evaluate the nursing students’ satisfaction with the conventional teaching method and the fused LSPPDM–simulation teaching method.

The self-learning ability scale of nursing students was compiled by Taiwan Su-Fen Cheng (13) and adapted by Wu Defang (14). The items and scores of this extensively used scale are widely assumed to accurately reflect students’ self-learning abilities. The scale includes four dimensions and a total of 20 items, with each dimension graded along a scoring interval of 1–5 points: 1 = strongly disagree, 2 = disagree, 3 = neutral, 4 = agree, and 5 = strongly agree. For comparative purposes, each of the 20 items was considered “qualified” when scored ≥3 on its 1–5 scale; thus, the overall scale ranged from 20 to 100 points, with 60 points representing the minimum acceptable total. The closer a student’s score is to 100, the stronger their capacity for autonomous learning; the closer their score to 0, the worse their capacity for autonomous learning. The Cronbach’s *α* coefficient of the total scale was 0.914.

Xu Juan’s (15) Chinese version of the Caring Ability Assessment Scale (CAI), an evaluation scale of humanistic care ability, was used. The scale assesses students’ caring ability along three aspects: cognition, courage, and patience, and includes 37 items with a total possible score of 259 points. The cognitive aspect contains 14 items with a total possible score of 98; the courage aspect contains 13 items with a total possible score of 91; and the patience aspect contains 10 items with a total possible score of 70. The cognitive and patience aspects are graded on an ascending scale contains 24 items, with a score range of 1–7 points. An item that is fully agreed with receives 7 points; an item that is absolutely opposed receives 1 point. The courage aspect is graded on a descending scale includes 13 items, and the scoring standard is 7–1 points. If the item is completely agreed with, it receives 1 point; if it is completely opposed, it receives 7 points. The higher a student’s score, the stronger their care ability. A total score above 220 points signifies a high caring ability, a total score between 203 and 220 points is indicative of an average level of caring ability, and a total score below 203 signifies a poor caring ability. The Cronbach’s *α* coefficient of the questionnaire in this population was 0.925, an indicator of good reliability and validity.

The Chinese registered nurse core competence scale (16) was used to evaluate the core competence of the nursing students in this study. The scale includes seven dimensions and 58 items: critical thinking (10 items), clinical nursing (9 items), leadership (10 items), interpersonal relationship (8 items), ethical/legal practice (8 items), professional development (6 items), and education/consultation (7 items). Each item is scored according to the Likert 5-level scoring method, with a score of 0–4 points and a total possible score of 232 points. A higher score indicates a stronger core nursing competence.

The evaluation of academic performance was calculated as follows: total score = process evaluation (30%) + final evaluation (70%). Process evaluation included three components: attendance rate, classroom participation, and homework (accounting for 30, 40, and 30% of the total process evaluation score, respectively), with the total possible score of each part being 100 points. Perfect attendance earned participants a score of 100 points, with each tardy incurring a 2-point deduction and each truancy a 5-point deduction. The maximum classroom participation score was 100 points. This included pre-class autonomous learning and in-class question answering, group discussion, and on-stage display, and was scored by teachers, with 10 points = excellent, 8 points = good, and 5 points = qualified. The homework score was also assigned by the teacher and included three possible entries: 10 points = excellent, 8 points = good, and 4 points = poor.

The theoretical examination questions of the summative evaluation were compiled by the teachers and included multiple choice, short answer, and Q&A questions, with a total possible score of 100 points. Practical skills were extracted according to training guidance and included materials preparation, operational steps, and training reports. The two groups adopted a unified scoring standard, with a total possible score of 100 points.

Based on a systematic literature review, we adapted items from an established teaching-satisfaction instrument and refined them to align with the specific objectives of the present study, thereby developing our evaluation form, and this included 4 teaching method items, 4 teaching effects items, and 2 teaching experience items.

### 2.4 Statistical analysis

Statistical software SPSS v. 26.0(IBM, Armonk, NY, United States) was used for statistical treatment. A normality test was conducted using the Kolmogorov–Smirnov test. Quantitative data up to normality was represented by mean ± standard deviation ( $\bar{x}$ ± *s*). A paired design *t* test was adopted for the comparison of the inter-group mean of paired differences; an independent sample *t* test was employed for group design. Counting data was represented by frequency number (*n*) and percentage (%); a chi-squared (*χ*^2^) test was used for those up to standard, and a Fisher’s exact test for those that were the substandard. Where bilateral *p* < 0.05, the differences were determined to be statistically significant.

To control for confounding variables, the following measures were taken. (1) Both groups of nursing students came from the same hospital and specialty and completed baseline questionnaires and theoretical and practical tests before their internships to confirm that no differences in academic performance or motivation existed between them. (2) Teaching faculty, class hours, textbooks, and assessment criteria were kept consistent to avoid differences in teacher competencies and resources. (3) Demographic variables such as gender, age, and educational level were included in covariance analysis to statistically control their influence on outcomes. (4) OSCEs were scored using a blinding method (examiners were unaware of group assignments) to reduce assessment bias. (5) Inclusion criteria were restricted to continuous internships of ≥8 months and absences of ≤1 week to exclude interferences arising from attendance and prior experience.

## 3 Results

### 3.1 General information comparison

In total, 71 student nurses (8 men, 63 women, aged 17.49 ± 0.62 y) were included in the observation group, and 72 student nurses (5 men, 67 women, aged 17.39 ± 0.56 y) were included in the control group. All data satisfied normality, and there were no statistically significant differences in sex, age, origin (urban vs. rural), or educational background between the two groups (*p* > 0.05; Table 2).

**Table 2:** Comparison of general information.

Item	Observation group (*n* = 71)	Control group (*n* = 72)	*χ^2^*/*t*	*p*
Age (years, x ± s)	17.49 ± 0.62	17.39 ± 0.56	1.051	0.295
Gender (M/F)	8/63	5/67	0.808	0.369
Place of Origin (n)	-	-	0.058	0.809
Urban	31	30	-	-
Rural	40	42	-	-
Only Child (n)	61	58	0.735	0.391
Education (n)	-	-	0.463	0.496
Specialized	52	49	-	-
Undergraduate	19	23	-	-
Serving as Class Committee in Class (n)	17	15	0.199	0.655
Reasons for Choosing Nursing Major (n)	-	-	1.885	0.597
Like It	31	30	-	-
Others’ Suggestions	20	23	-	-
Easy to Find a Job	12	15	-	-
Adjustment	8	4	-	-
Internship Duration (months, x ± s)	4.56 ± 2.46	5.73 ± 3.10	−0.642	0.531
Likeness to Nursing Major (n)	-	-	-	0.732
Like	22	19	-	-
General	48	51	-	-
Dislike	1	2	-	-

### 3.2 Comparison of self-learning ability between groups

There was no significant difference in learning motivation, planning and implementation, self-management, interpersonal communication, or total scores on the Self-Directed Learning Ability Scale between the two groups before training (*p* > 0.05). Learning motivation, planning and implementation, self-management, interpersonal communication, and total scores were higher in the observation group after training compared with the same metrics before training (*p* < 0.05). There was no significant difference in learning motivation, planning and implementation, self-management, interpersonal communication, or total scores in the control group before and after training (*p* > 0.05). After training, learning motivation (22.32 ± 3.41 vs. 20.41 ± 5.35; *t* = 2.240; *p* = 0.027), planning and implementation (19.71 ± 2.63 vs. 18.52 ± 3.52; *t* = 2.055; *p* = 0.042), self-management (14.25 ± 2.37 vs. 13.23 ± 3.38; *t* = 2.226; *p* = 0.028), interpersonal communication (14.11 ± 2.43 vs. 13.32 ± 1.78; *t* = 2.018; *p* = 0.045), and total scores (70.18 ± 7.11 vs. 66.45 ± 8.64; *t* = 3.503; *p* = 0.001) were higher in the observation group compared with the control group (Table 3).

**Table 3:** Comparison of self-learning ability between the two groups.

Item		Observation group (*n* = 71)	Control group (*n* = 72)	t value	*p* value	Cohen’s d	95% CI
Learning motivation	Before training	20.43 ± 4.50	20.31 ± 5.32	0.103	0.918	0.44	0.05–0.83
	After training	22.32 ± 3.41^a^	20.41 ± 5.35^b^	2.240	0.027		
Plan and implement	Before training	18.66 ± 3.71	18.33 ± 4.34	0.540	0.590	0.38	0.01–0.75
	After training	19.71 ± 2.63^a^	18.52 ± 3.52^b^	2.055	0.042		
Self-management	Before training	13.74 ± 4.19	12.35 ± 4.32	0.275	0.783	0.35	0.03–0.67
	After training	14.25 ± 2.37^a^	13.23 ± 3.38^b^	2.226	0.028		
Interpersonal communication	Before training	13.42 ± 2.46	13.24 ± 2.26	−0.044	0.965	0.36	0.01–0.71
	After training	14.11 ± 2.43^a^	13.32 ± 1.78^b^	2.018	0.045		
Total score	Before training	64.55 ± 9.11	65.47 ± 8.32	0.408	0.684	0.48	0.20–0.76
	After training	70.18 ± 7.11^a^	66.45 ± 8.64^b^	3.503	0.001		

a Compared with Before training, the difference was statistically significant.

b There was no significant difference between before training and after training.

### 3.3 Comparison of nursing student caring ability

There was no significant difference in cognition, courage, patience, or total scores on the Humanistic Care Scale between the two groups before training (*p* > 0.05). Courage, patience, and total scores were higher in the observation group after training compared with the same metrics before training (*p* < 0.05). There was no significant difference in cognition, courage, patience, or total scores of the control group before and after training (*p* > 0.05). After training, the cognition (48.31 ± 4.31 vs. 46.43 ± 6.23; *t* = 2.189; *p* = 0.030), courage (43.32 ± 4.13 vs. 41.43 ± 6.26; *t* = 2.352; *p* = 0.020), patience (35.24 ± 3.62 vs. 33.34 ± 5.24; *t* = 2.123; *p* = 0.035), and total scores (127.12 ± 4.23 vs. 121.28 ± 11.16; *t* = 3.997; *p* = 0.001) of the observation group were higher than those of the control group, as shown in Table 4.

**Table 4:** Comparison of caring ability of nursing students.

Item		Observation group (*n* = 71)	Control group (*n* = 72)	t value	*p* value	Cohen’s d	95% CI
Cognition	Before training	46.24 ± 6.32	46.34 ± 7.15	−0.016	0.987	0.36	0.04–0.68
	After training	48.31 ± 4.31^b^	46.43 ± 6.23^b^	2.189	0.030		
Courage	Before training	41.26 ± 6.16	41.45 ± 6.35	0.012	0.992	0.37	0.05–0.69
	After training	43.32 ± 4.13^a^	41.43 ± 6.26^b^	2.352	0.020		
Patience	Before training	33.34 ± 5.27	33.44 ± 5.34	0.507	0.613	0.34	0.02–0.66
	After training	35.24 ± 3.62^a^	33.34 ± 5.24^b^	2.123	0.035		
Total score	Before training	122.17 ± 14.46	121.23 ± 12.47	0.214	0.831	0.68	0.32–1.04
	After training	127.12 ± 4.23^a^	121.28 ± 11.16^b^	3.997	0.001		

a Compared with Before training, the difference was statistically significant.

b There was no significant difference between before training and after training.

### 3.4 Comparison of nursing student core competence

There was no significant difference in critical thinking ability, clinical nursing, leadership, interpersonal relationship, ethical/legal practice, professional development, education/consultation, or total scores between the two groups before training (*p* > 0.05). The critical thinking ability, clinical nursing, leadership, interpersonal relationship, ethical/legal practice, professional development, education/consultation, and total scores were higher in both groups after training compared with the same metrics before training (*p* < 0.05). After training, the critical thinking ability, clinical nursing, leadership, interpersonal relationship, ethics/legal practice, professional development, education/consultation, and total scores of the observation group were higher than those of the control group (*p* < 0.05), as shown in Table 5.

**Table 5:** Comparison of nursing core competence of nursing students.

Item		Observation group (*n* = 71)	Control group (*n* = 72)	*t* value	*p* value	Cohen’s d	95% CI
Critical thinking skills	Before training	15.75 ± 2.07	15.36 ± 2.02	0.739	0.463	0.77	0.25–1.29
	After training	23.47 ± 3.09^a^	21.21 ± 2.79^a^	2.973	0.004		
Clinical care	Before training	20.55 ± 2.71	20.26 ± 2.67	0.418	0.678	0.87	0.35–1.39
	After training	26.32 ± 3.47^a^	23.47 ± 3.09^a^	3.360	0.001		
Leadership	Before training	20.26 ± 2.67	20.77 ± 2.74	0.730	0.468	0.97	0.46–1.48
	After training	26.25 ± 3.46^a^	23.06 ± 3.04^a^	3.794	0.001		
Interpersonal relation	Before training	22.77 ± 3.12	22.65 ± 2.98	0.152	0.879	0.76	0.25–1.27
	After training	28.45 ± 3.75^a^	25.72 ± 3.39^a^	2.958	0.005		
Ethical/legal practice	Before training	16.06 ± 2.11	16.51 ± 2.17	0.814	0.419	1.20	0.67–1.73
	After training	22.77 ± 3.47^a^	19.06 ± 2.51^a^	4.745	<0.001		
Professional development	Before training	15.44 ± 2.03	15.06 ± 1.98	0.734	0.466	1.03	0.50–1.56
	After training	19.75 ± 2.62^a^	17.22 ± 2.27^a^	3.997	0.001		
Education/Consulting	Before training	12.06 ± 1.59	12.15 ± 1.65	0.215	0.830	0.69	0.17–1.21
	After training	16.32 ± 2.15^a^	14.89 ± 1.96^a^	2.692	0.009		
Total score	Before training	122.89 ± 16.22	122.76 ± 16.20	0.033	0.973	0.92	0.40–1.43
	After training	163.33 ± 21.55^a^	144.63 ± 19.09^a^	3.558	0.001		

a Compared with Before training, the difference was statistically significant.

### 3.5 Comparison of final academic performance

After training, classroom assessment (83.46 ± 4.41 vs. 81.32 ± 7.32; *t* = 2.252; *p* = 0.026), theoretical knowledge (81.85 ± 5.41 vs. 78.16 ± 10.87; *t* = 2.745; *p* = 0.007), practical skills (81.99 ± 7.36 vs. 78.1 ± 10.71; *t* = 2.700; *p* = 0.008), and final assessment scores (82.36 ± 3.35 vs. 79.09 ± 6.87; *t* = 3.860; *p* = 0.001) were higher in the observation group compared with those in the control group (Table 6).

**Table 6:** Comparison of final academic performance between the two groups of nursing students.

Item	Observation group (*n* = 71)	Control group (*n* = 72)	t value	*p* value	Cohen’s d	95% CI
Classroom assessment	83.46 ± 4.41	81.32 ± 7.32	2.252	0.026	0.36	0.04–0.68
Theoretical knowledge	81.85 ± 5.41	78.16 ± 10.87	2.745	0.007	0.43	0.12–0.74
Practice skills	81.99 ± 7.36	78.1 ± 10.71	2.700	0.008	0.42	0.11–0.73
Final assessment total score	82.36 ± 3.35	79.09 ± 6.87	3.860	0.001	0.61	0.29–0.93

### 3.6 Comparison of satisfaction with teaching model

After training, the teaching method (4.10 ± 0.58 vs. 3.42 ± 0.37; *t* = 7.486; *p* < 0.001), teaching effect (4.06 ± 0.58 vs. 3.27 ± 0.53; *t* = 8.593; *p* < 0.001), teaching experience (3.95 ± 0.71 vs. 3.33 ± 0.67; *t* = 6.305; *p* < 0.001), and total scores (12.03 ± 0.56 vs. 9.34 ± 0.35; *t* = 7.593; *p* < 0.001) of the observation group were higher than those of the control group (Table 7).

**Table 7:** Comparison of teaching satisfaction of nursing students.

Item	Observation group (*n* = 71)	Control group (*n* = 72)	*t* value	*p* value	Cohen’s d	95% CI
Teaching method	4.10 ± 0.58	3.42 ± 0.37	7.486	<0.001	1.40	1.01–1.79
Teaching efficiency	4.06 ± 0.58	3.27 ± 0.53	8.593	<0.001	1.42	1.08–1.76
Teaching experience	3.95 ± 0.71	3.33 ± 0.67	6.305	<0.001	0.89	0.61–1.17
Total score	12.03 ± 0.56	9.34 ± 0.35	7.593	<0.001	5.69	4.43–6.95

## 4 Discussion

Our results show that the experimental teaching practices used in this study (i.e., the combination LSPPDM–simulation teaching model) for pediatric nursing instruction possess significant advantages over more conventional practices. Students most need an ability to self-direct their learning (17, 18). Combining LSPPDM with simulation teaching methods can improve learning motivation, planning and execution, self-management, and interpersonal communication skills (18). Students’ inclination to actively participate in classroom activities greatly improves their language expression and interpersonal skills (19–21).

This study combined the LSPPDM training model with simulation-based teaching methods to instruct neonatal nursing interns. The results show that, with respect to self-directed learning ability, humanistic care ability, core neonatal nursing competencies, and academic performance, the observation group significantly outperformed the control group, indicating the significant pedagogical advantage of the combined model. Additional relevant literature, including successful case studies of LSPPDM in neonatal resuscitation technique training and empirical research on how simulation-based teaching enhances self-efficacy and clinical performance in nursing education, supports this conclusion. A meta-analysis of 12 randomized controlled trials showed that the standardized mean difference of the LSPPDM model in skill-based training was 0.78 (95% CI: 0.56–1.01), further bolstering the findings of this study (22).

The results of this study are consistent with the trends observed in two previous simulation-based teaching studies. Galehdar et al. (23) used scenario-based simulation combined with microteaching to train nursing students in pediatric basic life support, resulting in an increase in scores from 72.4 ± 8.1 to 86.3 ± 6.5, with an effect size of *d* = 1.12. Kasem et al. (24) utilized patients who had undergone standardization for mass casualty incident training, resulting in a 25% improvement in nursing students’ emergency decision-making scores. In this study, the combination LSPPDM–simulation teaching model improved neonatal nursing core competencies from 144.63 ± 19.09 to 163.33 ± 21.55 points, with an effect size of *d* = 0.92, and enhanced participants’ humanistic care and self-directed learning abilities, suggesting that this model has advantages in improving decision-making skills and emotional dimensions and can be extended to pediatric and emergency nursing courses.

Our research indicates that the integration of LSPPDM with simulation teaching significantly enhances the learning outcomes of neonatal nursing interns. The observation group in this study outperformed the control group in all assessments and teaching satisfaction metrics, with students universally acknowledging the model’s role in improving their learning efficiency, humanistic care, and professional identity. Its efficacy stems from the pre-instruction setting of teaching objectives, enabling students to engage in targeted learning, identify challenges through assessments, and foster self-reflection and improvement. However, a small number of students experienced reduced efficiency due to the excessive pressures of self-directed learning, highlighting the need for educators to prioritize personalized instruction, strengthen the supervision and guidance of self-directed learning, and provide timely support to those facing difficulties.

In addition, the observation group was found to have higher evaluation scores for various practical application results, as well as higher total teaching method scores, indicating that students accepted the combination LSPPDM–simulation teaching model (25). Compared with the control group, which used traditional teaching methods, the observation group showed greater satisfaction with the combination model’s effectiveness in improving their learning efficiency, humanistic care awareness, and professional identity.

From a theoretical perspective, LSPPDM aligns closely with constructivist learning theory in its promotion of students’ active knowledge construction through scenario simulation and reflective learning. Simulation-based teaching, grounded in self-efficacy theory, provides students with a safe practice environment to enhance their learning motivation and self-confidence. Additionally, the observation (S) and operation (D) stages of LSPPDM align with social learning theory, helping to improve students’ interpersonal communication skills. This integrated model demonstrates significant applicatory value in clinical practice, with the potential to enhance healthcare professionals’ clinical decision-making abilities and patient care quality. Despite challenges such as resource constraints and the authenticity of simulated scenarios, the integration of LSPPDM and simulation-based teaching will offer new opportunities and directions for nursing education reform as simulation technology advances and educational philosophies evolve.

This study has the following limitations: (1) its non-random grouping may have led to selection bias, such as differences in learning motivation or teacher quality between the two groups during their internships; (2) the small sample size and single-center nature of the study limits the generalizability of its results; (3) outcome measurements relied on self-reporting, which may introduce social desirability bias; and (4) the impact of differences in teachers’ instructional abilities on outcomes was not assessed. Future research should include multi-center, large-sample, randomized controlled trials to validate our findings.

## 5 Conclusion

In conclusion, the application of an LSPPDM–simulation teaching combination model to pediatric nursing teaching practices has significant advantages over conventional teaching models, with great potential for developing students’ self-directed learning ability, improving their humanistic caring capability, and strengthening their neonatal nursing core competence.

## Data Availability

The original contributions presented in the study are included in the article/supplementary material, further inquiries can be directed to the corresponding author.
